# The value of lactate dehydrogenase serum levels as a prognostic and predictive factor for advanced pancreatic cancer patients receiving sorafenib

**DOI:** 10.18632/oncotarget.5197

**Published:** 2015-09-10

**Authors:** Luca Faloppi, Maristella Bianconi, Riccardo Giampieri, Alberto Sobrero, Roberto Labianca, Daris Ferrari, Sandro Barni, Enrico Aitini, Alberto Zaniboni, Corrado Boni, Francesco Caprioni, Stefania Mosconi, Silvia Fanello, Rossana Berardi, Alessandro Bittoni, Kalliopi Andrikou, Michela Cinquini, Valter Torri, Mario Scartozzi, Stefano Cascinu, on behalf of the Italian Group for the Study of Digestive Tract Cancer (GISCAD)

**Affiliations:** ^1^ Medical Oncology Unit, Università Politecnica delle Marche, AOU “Ospedali Riuniti”, Ancona, Italy; ^2^ Medical Oncology Unit, Ospedale S. Martino, Genova, Italy; ^3^ Medical Oncology Unit, Ospedali Riuniti, Bergamo, Italy; ^4^ Medical Oncology Unit, Ospedale S. Paolo, Milano, Italy; ^5^ Medical Oncology Unit, Treviglio Hospital, Treviglio, Italy; ^6^ Medical Oncology Unit, C. Poma Hospital, Mantova, Italy; ^7^ Medical Oncology Unit, Fondazione Poliambulanza, Brescia, Italy; ^8^ Medical Oncology Unit, Arcispedale S. Maria Nuova IRCCS, Reggio Emilia, Italy; ^9^ New Drug Development Strategies Laboratory, Mario Negri Institute, Milano, Italy; ^10^ Medical Oncology Unit, Università degli Studi di Cagliari, Azienda Ospedaliero Universitaria, Cagliari, Italy

**Keywords:** pancreatic cancer, lactate dehydrogenase, angiogenesis, sorafenib, TKI

## Abstract

Although lactate dehydrogenase (LDH) serum levels, indirect markers of angiogenesis, are associated with a worse outcome in several tumours, their prognostic value is not defined in pancreatic cancer. Moreover, high levels are associated even with a lack of efficacy of tyrosine kinase inhibitors, contributing to explain negative results in clinical trials. We assessed the role of LDH in advanced pancreatic cancer receiving sorafenib.

Seventy-one of 114 patients included in the randomised phase II trial MAPS (chemotherapy plus or not sorafenib) and with available serum LDH levels, were included in this analysis. Patients were categorized according to serum LDH levels (LDH ≤vs.> upper normal rate).

A significant difference was found in progression free survival (PFS) and in overall survival (OS) between patients with LDH values under or above the cut-off (PFS: 5.2 vs. 2.7 months, *p* = 0.0287; OS: 10.7 vs. 5.9 months, *p* = 0.0021).

After stratification according to LDH serum levels and sorafenib treatment, patients with low LDH serum levels treated with sorafenib showed an advantage in PFS (*p* = 0.05) and OS (*p* = 0.0012).

LDH appears to be a reliable parameter to assess the prognosis of advanced pancreatic cancer patients, and it may be a predictive parameter to select patients candidate to receive sorafenib.

## INTRODUCTION

A growing body of evidence indicates that hypoxia may promote cancer development and it is involved in the resistance to treatment of cancer cells via the formation of new blood vessels.

Lactate dehydrogenase (LDH), is a key enzyme in the conversion of pyruvate to lactate under anaerobic conditions [1, 2]. The biological link between hypoxia, LDH levels and the tumor-driven angiogenesis pathway through the abnormal activation of the hypoxia inducible factor 1 (HIF-1) is well established. The biological activity of HIF-1 is determined by the expression and activity of the HIF-1α subunit [3]. HIF-1α is an essential factor that up-regulates a series of genes involved in glycolytic energy metabolism, angiogenesis, erythropoiesis and cell survival [4]. Hypoxia in the tumor microenvironment is sufficient to activate HIF-dependent expression of several down-regulated genes [5]. These include genes encoding for vascular endothelial growth factor, erythropoietin and many enzymes involved in glucose, iron, and nucleotide metabolism [6].

The role of hypoxia and how LDH may be useful to identify “hypoxic” tumours has been investigated by our group in colorectal carcinoma and hepatocellular carcinoma [7–9].

**Figure 1:**
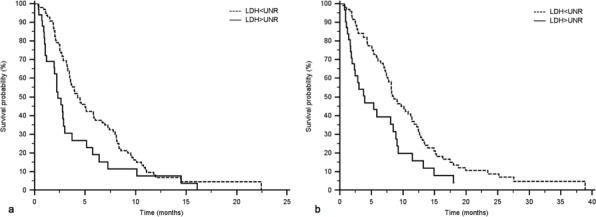
PFS (a) and OS (b) according to LDH serum values in a common practice population 132 advanced pancreatic cancer patients receiving a first line chemotherapy at our Institution from 2008 to 2012 were analysed. LDH ≤ UNR (99 patients) vs. LDH > UNR (33 patients): PFS 4.3 vs. 2.3 months, *p* = 0.0105; OS 8.6 vs. 3.9 months, *p* = 0.0042.

Recently, extending our analysis of LDH expression in pancreatic cancer patients, we have retrospectively evaluated the role of this serum marker in a common practice population of 132 advanced pancreatic cancer patients receiving a first line chemotherapy at our institution from 2008 to 2012. Results from this exploratory analysis have shown that LDH serum levels over the upper normal rate (UNR) were associated with poor prognosis in terms progression free survival (PFS) (Figure 1a) and overall survival (OS) (Figure 1b) (Table 1).

**Table 1:** Baseline patient characteristics of the common practice population evaluated in our exploratory analysis

LDH cut-off	LDH ≤ UNR		LDH > UNR		
**No of patients**	98		34		
**Median Age (range)**	65 (38–82)		62 (44–81)		
	*n*	%	*n*	%	*p*
**Treatment**					
Gemcitabine combination	79	81	25	73	0.383
Gemcitabine alone	19	19	9	27	
**Gender**					
Male	59	60	21	61	0.872
Female	39	40	13	39	
**Disease extent**					
Locally advanced	27	28	9	27	0.902
Metastatic	71	72	25	73	
**Primary tumour location**					
Head	57	58	22	65	0.502
Other	41	42	12	35	
**Biliary stenting**					
Yes	12	12	5	15	0.712
No	86	88	29	85	
**Metastatic sites location**					
Hepatic	47	48	15	45	0.698
Extra-hepatic	51	52	19	55	
**Metastatic site number**					
1	78	80	24	70	0.368
2	16	16	7	21	
3	3	3	1	3	
≥4	1	1	2	6	
Karnofsky PS*					
<70	8	8	5	15	0.269
≥70	90	92	29	85	

Furthermore, in preclinical studies, high levels of LDH were reported to predict resistance to several tyrosine kinase inhibitors (TKI), including sorafenib [10].

Based on these findings, after the encouraging results of our retrospective assessment, we decided to investigate the role of LDH in the phase II randomized trial (MAPS trial) assessing the role of Sorafenib, an anti-angiogenetic multitarget TKI, in combination with gemcitabine vs. gemcitabine alone in advanced pancreatic adenocarcinoma in order to find out a possible prognostic and predictive effect of LDH serum levels in this setting [11].

## MATERIALS AND METHODS

### Patients selection

All patients included in the “MAPS” phase randomized II trial with known LDH values were eligible for our analysis.

For all patients LDH values were collected within one month before the start of chemotherapy. We divided patients according to serum LDH levels in two groups: A: LDH ≤ upper normal rate (UNR) and B: LDH > UNR).

LDH serum levels were determined according to IFCC (International Federation of Clinical Chemistry and Laboratory Medicine) method. The assay has been conducted in Institution Laboratories certified for Quality control according to the present rules in Europe.

The follow-up and evaluation of treatment response protocols, applied in the MAPS phase two study, are summarized below.

Evaluations before and during treatment consisted of a complete medical history and physical examination; assessment of Karnofsky performance status; laboratory tests, including hematological and biochemical tests; CT or MRI of the abdomen or other body areas with disease involvement; and chest radiography or CT scan. Response Evaluation Criteria in Solid Tumors (RECIST) was used for defining response.

Assessments for response at each site were done blindly by a local experienced radiologist who was not directly involved in the trial.

Data validity was checked with periodical monitoring visits at the participating centers by the GISCAD group according to Good Clinical Practice (GCP) recommendations.

### Statistical analysis

The association between categorical variables was estimated by χ^2^ test.

Survival distribution was estimated by the Kaplan–Meier method (Kaplan and Meier, 1958).

Significant differences in probability of survival between the strata were evaluated by log-rank test.

A significant level of 0.05 was chosen to assess the statistical significance.

For statistical analysis, in both populations, median overall survival (mOS) and median progression free survival (mPFS) were defined as the interval between the date of beginning of treatment to death or last follow-up visit, and to clinical progression or death or last follow-up visit if not progressed.

## RESULTS

LDH values were available in 71 out of the 114 MAPS trial enrolled patients. Low LDH serum levels were present in 58 patients while 13 presented high LDH values.

**Figure 2:**
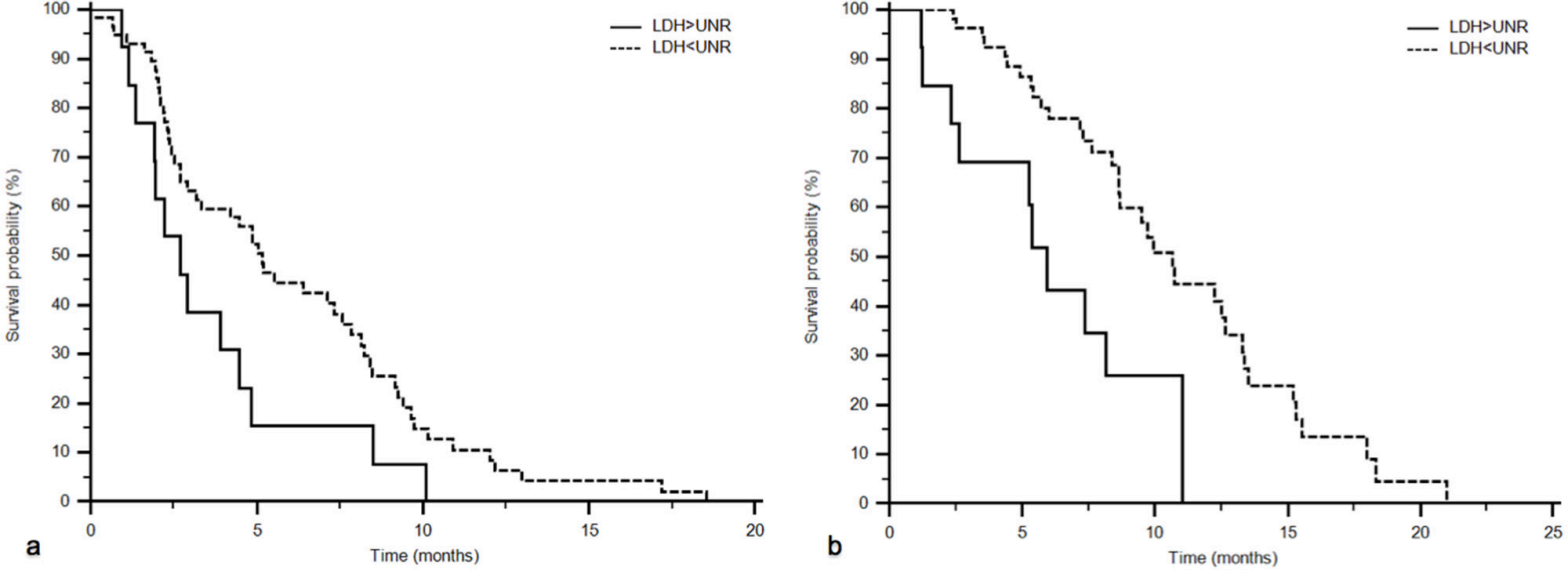
PFS (a) and OS (b) according to LDH serum values in the “MAPS” phase II study population LDH serum levels under or above the cut-off: PFS 5.2 vs. 2.7 months, *p* = 0.0287; OS 10.7 vs 5.9 months, *p* = 0.0021.

A statistically significant difference was found in PFS (Figure 2a) and in OS (Figure 2b) between patients with LDH values under (58 patients, 82%), or above (13 patients, 18%) the cut-off (group A vs. group B: mPFS 5.2 vs. 2.7 months, HR: 0.52, 95%CI: 0.24–1.11, *p* = 0.0287; mOS 10.7 vs 5.9 months, HR: 0.36, 95%CI: 0.13–0.98, *p* = 0.0021).

**Figure 3:**
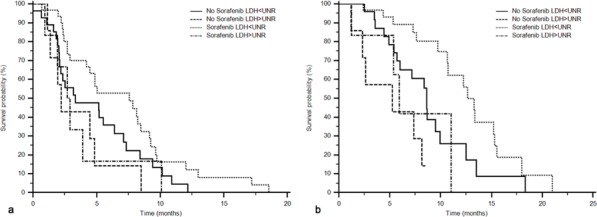
PFS (a) and OS (b) according to LDH serum values and treatment administered in the “MAPS” phase II study population PFS: sorafenib and LDH ≤ UNR: 31 patients, 7.6 months; sorafenib and LDH > UNR: 6 patients, 2.8 months; no sorafenib and LDH ≤ UNR: 27 patients, 3.3 months; no sorafenib and LDH > UNR: 7 patients, 2.2 months; *p* = 0.05. OS: sorafenib and LDH ≤ UNR: 12.7 months; sorafenib and LDH > UNR: 5.9 months; no sorafenib and LDH ≤ UNR: 8.6 months; no sorafenib and LDH > UNR: 5.2 months; *p* = 0.0012.

Stratifying the study population according to LDH serum levels and treatment (chemotherapy plus sorafenib or chemotherapy alone), patients with low LDH serum levels receiving sorafenib showed an advantage in PFS (Figure 3a; sorafenib and LDH ≤ UNR: 31 patients, 44%, 7.6 months; sorafenib and LDH > UNR: 6 patients, 8%, 2.8 months; no sorafenib and LDH ≤ UNR: 27 patients, 38%, 3.3 months; no sorafenib and LDH > UNR: 7 patients, 10%, 2.2 months; *p* = 0.05) and OS (Figure 3b; sorafenib and LDH ≤ UNR: 12.7 months; sorafenib and LDH > UNR: 5.9 months; no sorafenib and LDH ≤ UNR: 8.6 months; no sorafenib and LDH > UNR: 5.2 months; *p* = 0.0012) (Table 2).

**Table 2:** PFS and OS of “MAPS” phase II study population stratified according to LDH serum levels and treatment administered (chemotherapy plus sorafenib or chemotherapy alone)

Population subgroups		Patients number (%)	PFS (months)	OS (months)
Sorafenib group	LDH ≤ UNR	31 (44)	7.6	12.7
	LDH > UNR	6 (8)	2.8	5.9
No Sorafenib group	LDH ≤ UNR	27 (38)	3.3	8.6
	LDH > UNR	7 (10)	2.2	5.2
***p***			0.05	0.0012

The two patients groups proved homogeneous for all the clinical assessed variables (Table 3).

**Table 3:** Baseline patient characteristics in the subgroup of “MAPS” phase II trial population

LDH cut-off	LDH ≤ UNR		LDH > UNR		
No of patients (%)	58 (82)		13 (18)		
Median Age (range)	67 (45–77)		66 (46–81)		
	*n*	%	*n*	%	*p*
**Treatment**					
Sorafenib group	31	53	6	46	0.635
Non Sorafenib group	27	47	7	54	
**Gender**					
Male	32	55	9	69	0.354
Female	26	45	4	31	
**Disease extent**					
Locally advanced	20	34	5	38	0.786
Metastatic	38	66	8	62	
**Primary tumour location**					
Head	31	53	8	62	0.596
Other	27	47	5	38	
**Biliary stenting**					
Yes	5	9	2	15	0.459
No	53	91	11	85	
**Metastatic sites location**					
Hepatic	24	41	6	46	0.753
Extra-hepatic	34	59	7	54	
**Metastatic site number**					
1	51	88	9	69	0.119
2	5	9	2	15	
3	2	3	1	8	
≥4	0	0	1	8	
**Karnofsky PS**					
<70	16	28	4	31	
≥70	42	72	9	69	

## DISCUSSION

In last few years, cytotoxic and “biological” agents have resulted in no meaningful improvements in pancreatic cancer patients’ outcome[12, 13]. Several phase III studies assessing the role of targeted therapies, such as cetuximab [14], bevacizumab [15], erlotinib [16], aflibercept [17], and sorafenib [18], failed to show any significant benefit. One of the reasons of these negative results is the lack of patient selection.

Data from several analyses on different cancers seem to suggest that LDH levels may be a significant prognostic factor.

In colorectal cancer patients, LDH up-regulation was in fact associated with an increased risk of nodal and distant metastases and high LDH serum levels have been shown to correlate with a decreased median overall survival [19, 20].

A strong association between the expression of LDH and an aggressive phenotype has also been demonstrated in gastric cancer [21] and in hepatocellular carcinoma [7, 8, 22].

This apparently enhanced tumor aggressiveness, often determining a worse prognosis in cancer patients whit high LDH levels, have been correlated with molecular mechanisms underlying tumor hypoxia and angiogenesis. This possible link, LDH levels and tumor angiogenesis, has been analyzed in 2 different clinical trials (the CONFIRM 1 & 2 trials) investigating PTK/ZK (vatalanib), an oral VEGFR (vascular endothelial growth factor receptor) inhibitor in advanced colorectal cancer. Both these trials seemed to suggest that angiogenesis inhibitors are more effective in patients with high serum LDH levels, confirming the association between this serum marker and tumor angiogenesis [23, 24].

This evidence was confirmed in our work assessing the role of pre-treatment LDH serum levels in colorectal cancer patients receiving a first-line chemotherapy combined with bevacizumab, an anti-angiogenic monoclonal antibody. Bevacizumab showed an advantage in the subgroup of patients with high LDH levels and poor prognosis, confirming the predictive role of LDH in this setting [9].

However, it could be quite different using a TKI inhibitor, such as sorafenib. In fact we recently reported how high LDH serum levels in patients with hepatocellular carcinoma treated with sorafenib are associated with a worse outcome [8].

Based on these considerations, we reanalyzed our MAPS trial in order to evaluate the predictive and prognostic role of LDH serum levels. This analysis confirms the prognostic role of LDH serum levels. Of note, we were also able to show an advantage in PFS and OS in favour of sorafenib in low LDH serum levels, supporting a potential predictive value of LDH even for sorafenib. This clinical findings are supported by a preclinical model, where it has been demonstrated that the inhibition of LDH production with oxamic acid in cancer cell lines potentiated the antiproliferative activity of tyrosine kinase inhibitors, such as sorafenib [10]. The effect of high LDH levels on TKI low activity may be explained by a competition between ATP (adenosine triphosphate) and TKIs inhibition at the ATP enzymatic site on the protein kinases target of their activity.

LDH catalyzed the final step in the glycolytic pathway, the conversion of pyruvate and NADH to lactate and NAD^+^, determining the maintenance of glycolytic flow, and consequently, the production of ATP. In cancer cells, in hypoxic condition, in which anaerobic glycolysis is the main metabolic pathway to meet the energy request, the inhibition of LDH could interfere whit this process, causing the depletion of ATP and therefore a lower competition against TKIs inhibitors.

These preclinical findings support our hypothesis about the clinical role of LDH serum levels and are able to contribute to explain the negative results in our MAPS trial. Of interest could be a matching analysis of the results of BAYPAN phase III study [18].

The French authors in a similar trial with a comparable number of patients found a negative impact of sorafenib. Reanalyzing this trial according to LDH serum levels could open new perspectives for the use of sorafenib in pancreatic cancer patients.

Likewise data from a reanalysis of the CALGB-80303 phase III trial with bevacizumab could be interesting [15]. In this case we could observe that bevacizumab would be useful in patients with high LDH levels.

Probably, by assaying a single parameter such as LDH serum levels we could candidate patients to receive the best anti-angiogenetic treatment.

Although our study was not preplanned in the design of the MAPS trial and is conducted on a portion of the original cohort of patients, results are promising. Larger perspective studies focusing on LDH role are needed to confirm our findings.
